# Association between alternative healthy eating index (AHEI) with metabolic health status in adolescents with overweight and obesity

**DOI:** 10.1186/s12889-023-17558-8

**Published:** 2024-01-02

**Authors:** Donya Poursalehi, Ghazaleh Bahrami, Saeideh Mirzaei, Ali Asadi, Masoumeh Akhlaghi, Parvane Saneei

**Affiliations:** 1 Student Research Committee, Isfahan University of Medical Sciences, Isfahan, Iran; 2 Department of Community Nutrition, School of Nutrition and Food Science, Nutrition and Food Security Research Center, Isfahan University of Medical Sciences, PO Box 81745-151, Isfahan, Iran; 3 Department of Community Nutrition, School of Nutrition and Food Science, Shiraz University of Medical Sciences, Shiraz, Iran; 4 Department of Exercise Physiology, School of Physical Education and Sport Sciences, University of Tehran, Tehran, Iran

**Keywords:** Alternative healthy eating index, Metabolic health, Obesity, Adolescents

## Abstract

**Background:**

There has been lack of evidence on the association between healthy dietary patterns and metabolic health status of adolescents. The present study aimed to evaluate the association between alternative healthy eating index (AHEI) and metabolic health status among a relatively representative sample of Iranian adolescents with overweight/obesity.

**Methods:**

Adolescents with extra body weight (*n* = 203, aged 12–18 y), were selected for this cross-sectional study by a multistage cluster random-sampling method. Habitual dietary intakes and diet quality of individuals were assessed using validated food frequency questionnaire and AHEI-2010, respectively. Data on other covariates were also gathered by pre-tested questionnaires. To determine fasting glucose, insulin and lipid profiles, fasting blood samples were collected. Participants were categorized as having metabolically healthy overweight/obesity (MHO) or metabolically unhealthy overweight/obesity (MUO) phenotypes, based on two approaches (International Diabetes Federation (IDF) and combination of IDF with Homeostasis Model Assessment Insulin Resistance (HOMA-IR)).

**Results:**

The overall prevalence of MUO was 38.9% (based on IDF criteria) and 33.0% (based on IDF/HOMA-IR criteria). After considering all potential confounders, participants in highest tertiles of AHEI-2010 had lower odds of MUO profile according to both IDF (OR = 0.05; 95% CI: 0.01–0.15) and IDF/HOMA-IR (OR = 0.05; 95% CI: 0.02–0.19) definitions. This association was stronger in adolescents with overweight compared with obese ones and also among girls than boys. Moreover, each unit increase in AHEI-2010 score was associated with lower risk of MUO based on both criteria.

**Conclusions:**

Higher adherence to AHEI-2010 was inversely associated with odds of MUO in Iranian adolescents with overweight/obesity.

## Introduction

A significant increase in prevalence of overweight and obesity and its related comorbidities among adolescents over the past decades, both in early life and adulthood, have become major worldwide concerns [1]. The Center for Disease Control (CDC) has reported that about 1 in 5 American children and adolescents have obesity [2]. In 2016, more than 340 million children and adolescents (aged 5–19 years) around the globe had overweight/obesity [3]. Also, it has been predicted that by 2025, 413 million children and adolescents all around the world and 4 million children in Iran will have overweight/obesity [4, 5]. Overweight and obesity in early life are associated with serious health problems such as greater risk of cardiovascular diseases, type 2 diabetes, dyslipidemia and premature mortality [6, 7]. However not all subjects with overweight/obesity exhibit the mentioned complications.

Adolescents with metabolically healthy overweight/obesity (MHO) have a favourable cardio-metabolic status, presenting normal lipid profile, insulin sensitivity and blood pressure [8], while adolescents with metabolically unhealthy overweight/obesity (MUO) have an unfavourable metabolic profile [8]. These phenotypes could be converted to each other, suggesting that MHO individuals could become MUO over time [9]. Therefore, evaluating the influential factors including heredity, physical activity and diet would be critical to prevent this adverse transition [10, 11].

Dietary patterns could reflect the association between nutrition and metabolic health status more efficiently than individual food components [12]. As a result, many studies have evaluated the association of various dietary patterns such as Mediterranean diet, Dietary approaches to stop hypertension (DASH) and healthy eating patterns with metabolic disorders [13–15]. Adherence to a healthy dietary pattern determined by Alternative Healthy Eating Index-2010 (AHEI-2010) has been also associated with decreased odds of metabolic disturbances [16]. AHEI-2010 was developed to enhance assessment of dietary quality in relation to risk of chronic diseases [17]. The cumulative protective effects of AHEI-2010 components including fruits, vegetables and unsaturated fatty acids could justify the inverse association between this index and metabolic abnormalities [18].

A cross-sectional study on a sample of African-American adolescents showed that adherence to AHEI-2010 was associated with lower odds of metabolic syndrome (MetS) [19]. Another study conducted on American adolescents revealed an inverse association between AHEI-2010 and levels of hemoglobin A1C (Hb A1C); while, no significant association was observed between AHEI-2010 and odds of hypertension [20]. Also, no substantial relations between AHEI-2010 adherence and likelihood of cardiometabolic risk factors were found in a cross-sectional survey on both Brazilian and American adolescents [21]. Although previous studies provided information regarding the association between AHEI-2010 and metabolic disorders, there were limited data that evaluated this relationship among adolescents and in Middle-East countries, where dietary intakes differ from American and European countries. Also, to the best of our knowledge, no previous study has investigated AHEI-2010 in relation to metabolic health status, especially in adolescents. Therefore, the current study was conducted to evaluate the association of AHEI-2010 with metabolic health status in a sample of Iranian adolescents with overweight and obesity.

## Materials and methods

### Participants

A sample of Iranian adolescents aged 12 to 18 were included in this cross-sectional survey conducted in 2020. Considering previously published study, about 62.2 percent of adolescents with extra weight suffer from MUO profile [22]. Therefore, with a precision (d) of 7%, power of 80%, and type I error of 0.05 (confidence interval (CI) of 95%), a sample size of 185 participants were required to be minimally sufficient. Applying a multi-level cluster sampling method, a total of sixteen schools in different regions of Isfahan were randomly selected. Then, according to the standard growth curve of age-sex-specific body mass index (BMI) percentiles [23], students who had overweight and obesity were only invited to participate in our investigation. However, students were not included in the study if they 1) had a history of inherited or metabolic disorders including type 1 diabetes, hypothyroidism and Cushing's syndrome; 2) took supplements or medications which probably affect their metabolic profile; 3) followed a specific weight loss diet. Totally, 203 adolescents with overweight and obesity including 101 boys and 102 girls were included in our study. Participants filled out written consent forms after receiving information about the study. Informed consents were also collected from the parents of the participants, as minors were participated in the study. The study protocol adhered to the ethical principles outlined in the 1975 Declaration of Helsinki. The Local Ethics Committee of the Isfahan University of Medical Sciences (IUMS) approved the study protocol (no. IR.MUI.RESEARCH.REC.1402.116).

### Assessment of dietary intakes

We used a validated semi-quantitative food frequency questionnaire (FFQ) with 147 items to gather data on dietary intakes [24–26]. This FFQ has been previously used to reflect the association between dietary intakes of Iranian youths and a variety of disorders [27, 28]. Therefore, this questionnaire has satisfactory validity and reliability among Iranian adolescents [29]. Based on this FFQ, participants have determined the frequency of consumption (based on daily, weekly or monthly) and also amount of each food item during the previous year. Then, the portion size of eaten food was converted to gram per day using household measurements [30]. Finally, Nutritionist IV software, which has been modified for Iranian foods, was used to measure the amount of calorie and nutrients intake.

### Assessment of adherence to alternative healthy eating index-2010

AHEI-2010 consists of 11 components: fruits, vegetables, whole grains, nuts and legumes, long-chain n-3 fatty acids, polyunsaturated fatty acids (PUFA), red and processed meats, sugar-sweetened beverages and fruit juices, trans fatty acids, sodium and alcohol [17]. Due to a lack of relevant data in the current study, alcohol intake was excluded from the final score. AHEI-2010 was created by adjusting dietary intake of all components for total energy intake according to the residual approach [29]. Then, participants were divided into decile subgroups according to their energy-adjusted intakes of components. We utilized decile classifications as opposed to quantitative categories, since this approach would be less susceptible to misclassification. Individuals with the highest consumption of fruits, vegetables, whole grains, nuts and legumes, long-chain n-3 fatty acids and PUFA were given a score of 10 and those with the lowest consumption were scored 0. Conversely, other components including red and processed meats, sugar-sweetened beverages and fruit juices, trans fatty acids, and sodium were scored 0 in the highest consumption and 10 in the lowest consumption. The scores for the remaining deciles of these components were also allocated accordingly. The overall value of AHEI-2010 was determined by adding together the scores of all of these components, which ranged from 10 to 100.

### Assessment of metabolic health components

An experienced nutritionist assessed standing height and weight using a stadiometer (to the closest 0.1 cm) and calibrated electronic scale (to the nearest 0.1 kg), respectively. All anthropometric measurements were done while participants were wearing no shoes and as little clothing as possible. Furthermore, we calculated BMI based on Quetelet formula (weight (kg)/ height^2^ (m)) and categorized adolescents in terms of having overweight (85th < BMI < 95th percentile) or obesity (BMI > 95th percentile), according to World Health Organization (WHO) percentiles for age-sex-specific BMI [23]. Waist circumference (WC) measurement was taken to the nearest 0.1 cm, with a non-stretched, flexible tape after a typical expiration and with no external pressure applied. Blood pressure (BP) was measured while adolescents were sat on a chair with back supported and their feet were flat on the floor. Systolic blood pressure (SBP) and diastolic blood pressure (DBP) were assessed twice (after a 15-min break) by a mercury sphygmomanometer with a suitable cuff size, and the average BP was regarded in our analysis. Blood samples were taken from participants in a twelve-hour fasting state in the laboratory and then, blood samples were centrifuged at 3500 rpm within 10 min to separate the serum. Concentrations of fasting blood glucose (FBG), triglycerides (TG), high density lipoprotein cholesterol (HDL-c) and insulin were evaluated on the day of sampling, using glucose oxidase, glycerol phosphate oxidase, cholesterol oxidase, and electrochemiluminescence immunoassay methods, respectively. Homeostasis Model Assessment Insulin Resistance (HOMA-IR) formula was also applied to determine Insulin resistance (IR) [31]: [(fasting insulin (mU/L) × FBG (mmol/L)]/22.5.

### Metabolic status definitions

Two different approaches were used to distinguish MHO and MUO phenotypes. In the first method, metabolic health status was determined based on the International Diabetes Federation (IDF) [32]. Such that, individuals were considered to be MUO if they had two or more of the following risk factors: 1) elevated FBG (≥ 100 mg/dL), 2) increased TG (≥ 150 mg/dL), 3) reduced HDL-c (< 40 mg/dL for the age of < 16 y, and < 50 mg/dL for girls/ < 40 mg/dL for boys in the ages of ≥ 16 y), 4) increased BP (≥ 130/85 mmHg). On the other hand, adolescents with less than two of mentioned items were recognized as MHO. In the second strategy, existence of IR was also assessed in addition to IDF [33]. Therefore, if participants had at least two items of IDF criteria and HOMA-IR scores ≥ 3.16, they were assumed to be MUO and those with HOMA-IR < 3.16 were considered a MHO.

### Assessment of other variables

Socioeconomic status (SES) of participants was assessed through a validated questionnaire including following items: parental education level, parental job, family size, having a personal room, traveling in a year, number of cars and laptops/computers in the family [34]. Data on age, gender, medications or supplements intake and history of diseases were also gathered using another self-administered questionnaire. A validated Physical Activity Questionnaire specified for Adolescents (PAQ-A) was applied to estimate physical activity (PA) level of participants [35]. This questionnaire includes nine items that evaluates usual and unusual activities of subjects over the previous week. By calculating total score, adolescents were categorized into four groups: sedentary (score < 2), low-active (3 < score ≤ 2), active (score ≥ 3), and very active (score ≥ 4). Due to the small number of sedentary and very active participants, we merged sedentary with low-active and active with very active to create two final categories: low level vs. high level.

### Statistical analysis

Statistical analysis was done using SPSS software version 26 (IBM, Chicago, IL). The Kolmogorov–Smirnov test was used to assess the normal distribution of quantitative data. Participants were classified based on tertiles of energy-adjusted AHEI-2010. General characteristics of adolescents were expressed as percentage for qualitative variables and mean ± SD/SE for quantitative variables. We used one-way analysis of variance (ANOVA) and chi-square test to respectively assess continuous and categorical variables across tertiles of AHEI-2010. Age-, gender- and energy-adjusted dietary intakes of participants across tertiles of AHEI-2010 were also assessed using analysis of covariance (ANCOVA). To determine the association between AHEI-2010 with MUO, binary logistic regression was utilized in different models. First, the confounding effects of age, gender and energy intake were considered. In the second model, additional adjustments were done for SES and PA. Lastly, BMI was additionally controlled as a confounder. Stratified analyses were conducted based on BMI categories (overweight vs. obese) and sex (boys vs. girls). Moreover, we regarded AHEI-2010 tertiles as an ordinal variable to compute the trend of odds ratios (ORs) over increasing AHEI-2010 tertiles. In all analyses, the first tertile of AHEI-2010 was inputted as the reference group. AHEI-2010 was additionally employed as a continuous variable to assess a linear relation. *P*-values < 0.05 were accepted to be significant.

## Results

Main characteristics of participants are summarized in Table 1. The study sample consisted of 102 girls and 101 boys aged 12–18 years old that all had overweight or obesity (mean weight: 73.48 ± 11.60 kg). The average AHEI-2010 score was 55.00 ± 13.07. Compared with the first tertile of AHEI-2010, adolescents in the last tertile had lower weight, BMI, WC, SBP, DBP, FBS, Insulin, HOMA-IR, TG and HDL-c. Conversely, they were more likely to be physically active. However, age, gender and SES did not differ noticeably among categories of AHEI-2010.

**Table 1:** General characteristics of study participants across tertiles of the alternate healthy eating index-2010 (*n* = 203)^1^

	Tertiles of energy-adjusted alternate healthy eating index-2010^2^			
	T_1_ (*n* = 68) Score < 49	T_2_ (*n* = 67) 49–62	T_3_ (*n* = 68) > 62	*P*^3^
Alternate healthy eating index-2010	40.16 ± 5.87	55.25 ± 4.08	69.60 ± 4.89	< 0.001
Age (y)	14.03 ± 1.54	13.87 ± 1.52	14.4 ± 1.77	0.78
Weight (kg)	75.65 ± 10.67	74.13 ± 11.88	70.68 ± 11.83	0.04
Body mass index (kg/m^2^)	27.76 ± 2.65	27.79 ± 3.43	26.52 ± 3.46	0.03
Overweight/obesity prevalence, n (%)				< 0.001
Overweight	25 (36.8)	30 (44.8)	49 (72.1)	
Obese	43 (63.2)	37 (55.2)	19 (27.9)	
Waist circumference (cm)	91.68 ± 7.03	91.65 ± 7.16	87.68 ± 8.91	0.01
Sex, n (%)				0.68
Boy	31 (45.6)	34 (50.7)	36 (52.9)	
Girl	37 (54.4)	33 (49.3)	32 (47.1)	
Physical activity levels, n (%)				< 0.001
Low^4^	67 (98.5)	63 (94)	36 (52.9)	
High^5^	1 (1.5)	4 (6.0)	32 (47.1)	
Socioeconomic status levels, n (%)				0.53
Low	24 (35.3)	18 (26.9)	17 (25)	
Moderate	30 (44.1)	28 (41.8)	32 (47.1)	
High	14 (20.6)	21 (31.3)	19 (27.9)	
Systolic blood pressure (mmHg)	116.71 ± 10.41	113.69 ± 21.83	107.74 ± 19.88	0.01
Diastolic blood pressure (mmHg)	76.29 ± 5.95	73.41 ± 13.04	73.50 ± 11.38	0.02
Fasting blood sugar (mg/dL)	102.38 ± 8.92	98.60 ± 7.58	93.43 ± 6.41	< 0.001
Insulin (µUI/mL)	23.78 ± 14.67	21.05 ± 10.31	16.44 ± 11.63	0.01
HOMA-IR index	6.00 ± 3.60	5.22 ± 2.87	3.85 ± 2.98	< 0.001
Triglycerides (mg/dL)	142.75 ± 62.79	125.19 ± 76.63	97.96 ± 50.94	< 0.001
HDL cholesterol (mg/dL)	42.31 ± 7.91	44.49 ± 7.00	47.66 ± 7.98	< 0.001

^1^All values are means ± standard deviation (SD), unless indicated

^2^Alternate healthy eating index-2010 components were adjusted for energy intake based on residual method

^3^Obtained from ANOVA for continuous variables and chi-square test for categorical variables

^4^Includes sedentary and low-active participants

^5^Includes active and very-active participants

Table 2 indicates dietary intakes of participants across tertiles of AHEI-2010. Individuals with the highest adherence to AHEI-2010 consumed more amounts of fruits, vegetables, legumes, nuts, whole grains and lower amounts of red and processed meats, sugar-sweetened beverages and fruit juices than those with the least adherence. In case of nutrients, individuals with the highest AHEI-2010 adherence had also higher intake of protein and lower intake of saturated and trans fatty acids. However, no significant differences were observed between intakes of fats, carbohydrates, cholesterols, monounsaturated and polyunsaturated and omega-3 fatty acids across AHEI-2010 tertiles.

**Table 2:** Multivariable-adjusted intakes of selected food groups and nutrients of study participants across tertiles of Alternate Healthy Eating Index-2010 (*n* = 203)^1^

	Tertiles of energy-adjusted alternate healthy eating index-2010^2^			
	T_1_ (*n* = 68) Score < 49	T_2_ (*n* = 67) 49–62	T_3_ (*n* = 68) > 62	*P*^3^
Energy (Kcal/d)	2949.90 ± 65.84	2864.51 ± 66.31	2834.39 ± 65.86	0.44
**Food groups:**				
Fruits (g/day)	219.35 ± 16.23	302.40 ± 16.31	363.15 ± 16.22	< 0.001
Vegetables (g/day)	168.62 ± 18.26	279.16 ± 18.34	380.43 ± 18.24	< 0.001
Red and processed meats (g/day)	35.89 ± 1.72	27.83 ± 1.73	15.42 ± 1.72	< 0.001
Legumes (g/day)	33.43 ± 3.34	55.44 ± 3.35	57.96 ± 3.33	< 0.001
Nuts (g/day)	7.94 ± 1.25	11.12 ± 1.26	17.47 ± 1.25	< 0.001
Whole grains (g/day)	178.46 ± 13.46	227.44 ± 13.53	276.27 ± 13.45	< 0.001
Sugar-sweetened beverages and fruit juices (g/day)	113.57 ± 6.18	72.37 ± 6.21	41.98 ± 6.18	< 0.001
**Other nutrients:**				
Proteins (% of energy)	13.71 ± 0.24	14.41 ± 0.24	14.81 ± 0.24	0.01
Fats (% of energy)	28.86 ± 0.63	28.64 ± 0.64	29.04 ± 0.64	0.91
Carbohydrates (% of energy)	58.67 ± 0.63	58.48 ± 0.64	57.74 ± 0.63	0.55
Cholesterol (mg/d)	286.88 ± 11.93	268.04 ± 11.99	291.09 ± 11.92	0.35
Saturated fats (mg/d)	28.81 ± 0.70	26.61 ± 0.70	26.63 ± 0.70	0.04
Trans fatty acids (g/d)	8.49 ± 0.36	6.00 ± 0.36	3.26 ± 0.36	< 0.001
Monounsaturated fatty acids (g/d)	27.80 ± 0.84	26.97 ± 0.84	27.87 ± 0.84	0.70
Polyunsaturated fatty acids (g/d)	27.11 ± 0.96	28.33 ± 0.97	30.02 ± 0.96	0.11
Long chain n-3 fatty acids (g/d)	0.58 ± 0.02	0.67 ± 0.02	0.59 ± 0.02	0.16
Sodium (mg/d)	4566.09 ± 129.33	3910.78 ± 129.94	3488.25 ± 129.22	< 0.001

^1^All values are means ± standard error (SE); energy and macronutrients intake is adjusted for age and sex; all other values are adjusted for age, sex and energy intake

^2^Alternate healthy eating index-2010 components were adjusted for energy intake based on residual method

^3^Obtained from ANCOVA

**Fig. 1:**
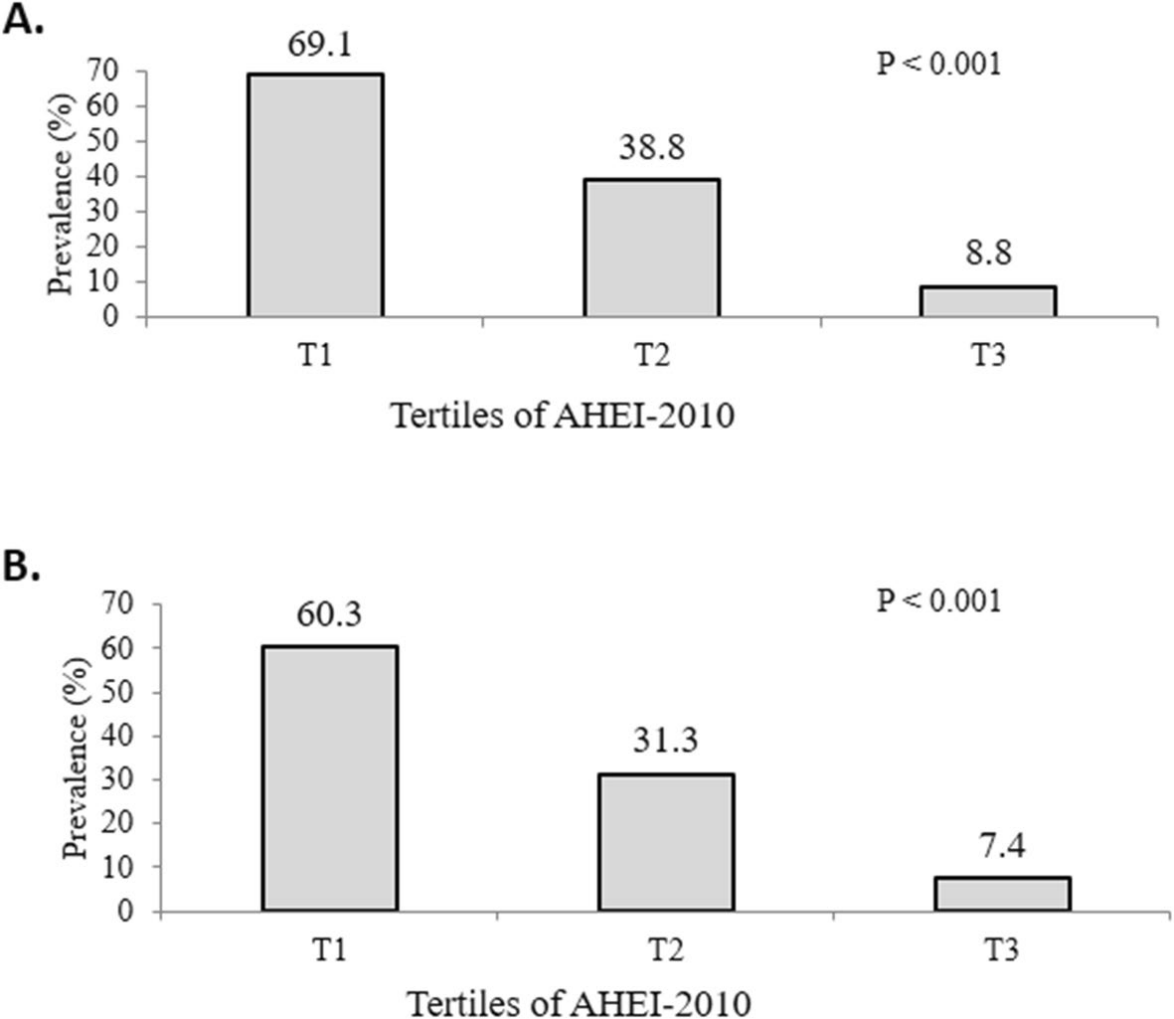
Prevalence of MUO in energy-adjusted tertiles of Alternate Healthy Eating Index-2010. **A** MUO Based on IDF definition. **B** MUO Based on IDF/HOMA-IR IDF definition. Values are percentage of adolescents with a metabolically unhealthy profile in tertiles of Alternate Healthy Eating Index-2010. *P*-values were obtained from chi-square test

Overall prevalence of MUO was 38.9% according to IDF criteria and 33.0% based on IDF/HOMA-IR criteria. Prevalence of MUO across tertiles of AHEI-2010 is illustrated in Fig. 1. Compared with the lowest category, individuals with the highest adherence to AHEI-2010 had a lower prevalence of MUO based on IDF definition (T3 vs. T1: 8.8% vs. 69.1%; *P* < 0.001). Same finding was found based on IDF/HOMA-IR criteria (T3 vs. T1: 7.4% vs. 60.3%; *P* < 0.001).

Crude and multivariable-adjusted ORs for MUO among tertiles of AHEI-2010 are represented in Table 3. According to IDF method, a higher score of AHEI-2010 was associated with decreased chance of MUO profile in the crude model (OR_T3 vs. T1_ = 0.04; 95% CI: 0.02–0.12). This association remained unchanged after considering probable cofounders (OR_T3 vs. T1_ = 0.05; 95% CI: 0.01–0.15). Based on IDF/HOMA-IR definition, an inverse association was also observed between AHEI-2010 adherence and MUO in both crude (OR_T3 vs. T1_ = 0.05; 95% CI: 0.02–0.15) and multivariable-adjusted (OR_T3 vs. T1_ = 0.05; 95% CI: 0.02–0.19) models. Furthermore, by increasing AHEI-2010 tertiles, a downtrend in MUO profile was observed (P_trend_ < 0.001 for all models). Each unit increment in AHEI-2010 score was additionally related to decreased odds of MUO based on IDF and IDF/HOMA-IR criteria.

**Table 3:** Multivariable-adjusted odds ratio for MUO across tertiles of alternate healthy eating index-2010 (*n* = 203)^a^

	Tertiles of energy-adjusted alternate healthy eating index-2010^b^				Per 1 score (unit) increase in AHEI-2010
	T_1_ (*n* = 68) Score < 49	T_2_ (*n* = 67) 49–62	T_3_ (*n* = 68) > 62	P_trend_	
***MUO Based on IDF criteria***					
MUO cases (n)	47	26	6		
Crude	1.00	0.28 (0.14, 0.58)	0.04 (0.02, 0.12)	< 0.001	0.91 (0.88, 0.94)
Model 1	1.00	0.31 (0.15, 0.63)	0.04 (0.01, 0.12)	< 0.001	0.91 (0.88, 0.94)
Model 2	1.00	0.34 (0.16, 0.71)	0.05 (0.02, 0.15)	< 0.001	0.91 (0.88, 0.95)
Model 3	1.00	0.32 (0.15, 0.68)	0.05 (0.01, 0.15)	< 0.001	0.91 (0.88, 0.94)
***MUO Based on IDF/HOMA-IR criteria***					
MUO cases (n)	41	21	5		
Crude	1.00	0.30 (0.15, 0.61)	0.05 (0.02, 0.15)	< 0.001	0.91 (0.89, 0.94)
Model 1	1.00	0.31 (0.15, 0.66)	0.05 (0.02, 0.15)	< 0.001	0.91 (0.88, 0.94)
Model 2	1.00	0.35 (0.16, 0.74)	0.06 (0.02, 0.20)	< 0.001	0.92 (0.89, 0.95)
Model 3	1.00	0.32 (0.15, 0.69)	0.05 (0.02, 0.19)	< 0.001	0.92 (0.88, 0.95)

^a^All values are odds ratios and 95% confidence intervals. Model 1: Adjusted for age, sex, energy intake. Model 2: More adjustments for physical activity levels, socioeconomic status. Model 3: Further adjustments for BMI

^b^Alternate healthy eating index-2010 components were adjusted for energy intake based on residual method

ORs for MUO across tertiles of AHEI-2010 stratified by BMI categories and sex are shown in Tables 4 and 5, respectively. Based on IDF definition, greater adherence to AHEI-2010 was significantly associated with reduced odds of MUO in both groups of adolescents with overweight (adjusted-OR_T3 vs. T1_ = 0.02; 95% CI: 0.01–0.12) and obesity (adjusted-OR_T3 vs. T1_ = 0.15; 95% CI: 0.04–0.63). Considering IDF/HOMA-IR method, the same findings were observed in subjects with overweight (adjusted-OR_T3 vs. T1_ = 0.04; 95% CI: 0.01–0.37) and obesity (adjusted-OR_T3 vs. T1_ = 0.10; 95% CI: 0.02–0.48) (Table 4). Among girls, those in the top tertile of AHEI-2010 in comparison with the bottom tertile had a lower chance of MUO profile according to both IDF (adjusted-OR_T3 vs. T1_ = 0.02; 95% CI: 0.01–0.11) and IDF/HOMA-IR (adjusted-OR_T3 vs. T1_ = 0.02; 95% CI: 0.01–0.23) criteria. Similarly, an inverse association was observed between higher AHEI-2010 score and MUO in boys; however, the magnitude of this association was weaker than in girls [based on IDF definition: fully-adjusted OR_T3 vs. T1_ = 0.05; 95% CI: 0.01–0.29; based on IDF/HOMA-IR definition: fully-adjusted OR_T3 vs. T1_ = 0.05; 95% CI: 0.01–0.34] (Table 5).

**Table 4:** Multivariable-adjusted odds ratio for MUO across tertiles of alternate healthy eating index-2010 stratified by BMI categories (*n* = 203)^a^

	Tertiles of energy-adjusted alternate healthy eating index-2010^b^			
	T_1_ (*n* = 68) Score < 49	T_2_ (*n* = 67) 49–62	T_3_ (*n* = 68) > 62	P_trend_
***MUO Based on IDF criteria***				
***Overweight (cases/participants)***	17/25	10/30	1/49	
Crude	1.00	0.24 (0.08, 0.73)	0.02 (0.01, 0.08)	< 0.001
Model 1	1.00	0.26 (0.08, 0.85)	0.02 (0.01, 0.09)	< 0.001
Model 2	1.00	0.30 (0.09, 1.03)	0.02 (0.01, 0.12)	< 0.001
***Obesity (cases/participants)***	30/43	16/37	5/19	
Crude	1.00	0.33 (0.13, 0.83)	0.16 (0.05, 0.52)	0.01
Model 1	1.00	0.34 (0.13, 0.88)	0.13 (0.04, 0.50)	0.01
Model 2	1.00	0.37 (0.14, 0.97)	0.15 (0.04, 0.63)	0.01
***MUO Based on IDF/HOMA-IR criteria***				
***Overweight (cases/participants)***	12/25	7/30	1/49	
Crude	1.00	0.33 (0.10, 1.05)	0.02 (0.01, 0.19)	< 0.001
Model 1	1.00	0.33 (0.10, 1.14)	0.02 (0.01, 0.20)	< 0.001
Model 2	1.00	0.39 (0.11, 1.41)	0.04 (0.01, 0.37)	0.01
***Obesity (cases/participants)***	29/43	14/37	4/19	
Crude	1.00	0.29 (0.12, 0.74)	0.13 (0.04, 0.46)	0.01
Model 1	1.00	0.31 (0.12, 0.79)	0.10 (0.03, 0.43)	0.01
Model 2	1.00	0.32 (0.12, 0.84)	0.10 (0.02, 0.48)	0.01

^a^All values are odds ratios and 95% confidence intervals. Model 1: Adjusted for age, sex, energy intake. Model 2: More adjustments for physical activity levels, socioeconomic status

^b^Alternate healthy eating index-2010 components were adjusted for energy intake based on residual method

**Table 5:** Multivariable-adjusted odds ratio for MUO across tertiles of alternate healthy eating index-2010 stratified by sex (*n* = 203)^a^

	Tertiles of energy-adjusted alternate healthy eating index-2010^b^			
	T_1_ (*n* = 68) Score < 49	T_2_ (*n* = 67) 49–62	T_3_ (*n* = 68) > 62	P_trend_
***MUO Based on IDF criteria***				
***Girls (cases/participants)***	27/37	13/33	2/32	
Crude	1.00	0.24 (0.09, 0.66)	0.03 (0.01, 0.12)	< 0.001
Model 1	1.00	0.24 (0.08, 0.72)	0.02 (0.01, 0.09)	< 0.001
Model 2	1.00	0.24 (0.08, 0.73)	0.02 (0.01, 0.12)	< 0.001
Model 3	1.00	0.24 (0.08, 0.75)	0.02 (0.01, 0.11)	< 0.001
***Boys (cases/participants)***	20/31	13/34	4/36	
Crude	1.00	0.34 (0.12, 0.94)	0.07 (0.02, 0.25)	< 0.001
Model 1	1.00	0.37 (0.13, 1.02)	0.07 (0.02, 0.25)	< 0.001
Model 2	1.00	0.47 (0.16, 1.38)	0.08 (0.02, 0.41)	0.01
Model 3	1.00	0.41 (0.13, 1.25)	0.05 (0.01, 0.29)	0.01
***MUO Based on IDF/HOMA-IR criteria***				
***Girls (cases/participants)***	22/37	9/33	1/32	
Crude	1.00	0.26 (0.09, 0.70)	0.02 (0.01, 0.18)	< 0.001
Model 1	1.00	0.22 (0.07, 0.69)	0.02 (0.01, 0.15)	< 0.001
Model 2	1.00	0.22 (0.07, 0.70)	0.02 (0.01, 0.21)	< 0.001
Model 3	1.00	0.22 (0.07, 0.70)	0.02 (0.01, 0.23)	< 0.001
***Boys (cases/participants)***	19/31	12/34	4/36	
Crude	1.00	0.34 (0.13, 0.94)	0.08 (0.02, 0.28)	< 0.001
Model 1	1.00	0.37 (0.13, 1.04)	0.08 (0.02, 0.30)	< 0.001
Model 2	1.00	0.49 (0.16, 1.46)	0.10 (0.02, 0.47)	0.01
Model 3	1.00	0.43 (0.14, 1.32)	0.05 (0.01, 0.34)	0.01

^a^All values are odds ratios and 95% confidence intervals. Model 1: Adjusted for age, energy intake. Model 2: More adjustments for physical activity levels, socioeconomic status. Model 3: Further adjustments for BMI

^b^Alternate healthy eating index-2010 components were adjusted for energy intake based on residual method

## Discussion

In this cross-sectional study, we found a strong negative association between scores of AHEI-2010 and odds of MUO based on IDF and IDF/HOMA-IR criteria. After considering probable cofounders, this association remained unchanged. This relation was stronger in adolescents with overweight compared with obesity and also in girls than boys. Furthermore, each unit increment in AHEI-2010 score was related to decreased odds of MUO.

Obesity acts as a multisystem disorder that is associated with progression of different comorbidities such as cardiometabolic disease, cancers, mental disorders and even death [36]. Excessive fat accumulation and its subsequent consequences, especially among adolescents, are of the major challenges of both developed and developing countries which impose a lot of costs on healthcare systems [37]. So, it is imperative to control overweight/obesity and prevent progressing MHO to MUO status. Our findings showed an inverse association of AHEI-2010 and MUO profiles among adolescents. Hence, more adherence to AHEI-2010 and its components could be a worthwhile clinical advice for adolescents to prevent emerging metabolic comorbidities of obesity.

The Healthy Eating Index (HEI) has been created to assess diet quality based on dietary guidelines for Americans [38]. Several modified HEIs have been measured in relation to metabolic disorders. In a cross-sectional study among American adolescents, adherence to HEI-2015 was in association with decreased odds of MetS [39]. However, no substantial relation was concluded from another study with HEI-2010 and cardiometabolic risk factors [40]. A cross-sectional investigation on 706 Iranian adolescents illustrated that higher consistency with HEI-2005 was associated with lower odds of hypertriglyceridemia and low serum levels of HDL-c; whereas, no significant association was observed with MetS [41]. In our study, AHEI-2010 index was utilized to evaluate diet quality of participants in relation to odds of MUO. Decimal scoring in this index would reduce the possibility of misclassification and enhance the accuracy of our analyses.

We found an inverse association between AHEI-2010 adherence and odds of MUO. In line with our finding, a prospective study conducted among Iranian adults demonstrated that greater adherence to AHEL-2010 was associated with reduced odds of MetS [42]. In addition, a prior cross-sectional study on a sample of adults with obesity revealed an inverse relationship between AHEI-2010 adherence and likelihood of hyperglycemia and hypertriglyceridemia [43]. Another cross-sectional investigation among Canadian adults showed that individuals with higher scores of AHEI-2010 had lower risk of developing MetS [44]. However, there is a lack of data relating AHEI-2010 to metabolic disorders in children and adolescents. Ducharme-Smith et al. demonstrated an inverse relationship between greater AHEI-2010 scores and odds of MetS in adolescents [19]. In another study among adolescents aged 10–19 years, AHEI-2010 adherence was in association with low levels of Hb A1C, but not with hypertension [20]. Furthermore, AHEI-2010 adherence was not associated with reduced odds of cardiometabolic risk factors in a cross-sectional study on 189 Brazilian and 787 Hispanic/Latino American adolescents [21]. These inconsistencies between findings of the mentioned studies could be attributed to the differences in study designs, populations, measurement tools for assessing dietary intakes, metabolic disorders, and also different confounding variables in the analyses. It is worth noting that our study was conducted among adolescents with overweight/obesity, while most previous studies were performed among individuals with different BMI categories.

In the current analysis, adherence to AHEI-2010 has stronger association with MUO among adolescents with overweight than obesity. Although adopting a healthy lifestyle, particularly with regard to diet is beneficial for both overweight and obesity conditions, metabolic state of the body in overweight might be more sensitive to these factors to overcome the detrimental health consequences of extra weight [45]. Moreover, the strength of the observed association was greater in girls than boys. Different dietary habits may be the reason behind this finding in which, girls prefer higher intake of fruits and vegetables and lower intake of fatty and processed products [46]. In addition, girls often have a healthier metabolic profile that could be due to the differences in hormonal status and distribution of extra fat throughout their bodies [47]. Nevertheless, differences in number of cases and also range of AHEI-2010 in each tertile should not be ignored.

The fundamental cause of positive relation between AHEI-2010 and healthier metabolic status is not exactly clear and needs further studies. However it is well known that AHEI-2010 is accompanied by higher intakes of fruits, vegetables, whole grains, legumes and nuts which contain high amounts of fiber. Dietary fiber intake could decrease abdominal obesity by regulating energy homeostasis. Fiber intake could also reduce glycemic response and have cholesterol-lowering effects [48]. In addition, this index is restricted in consumption of red and processed meats that has substantial role in increasing obesity-related inflammatory markers [49]. Due to the documented association of inflammation and metabolic unhealthy status [50], reduction in intake of red and processed meat could prevent metabolic disorders development. Moreover, lower intake of carbohydrates, specifically from refined sources, could result in elevated HDL-c and decreased serum TG levels which are directly associated with lower odds of metabolic disorders [51].

The present study has several strengths and weaknesses. This is the first study exploring the association of AHEI-2010 with metabolic health status in a sample of Iranian adolescents with overweight and obesity. Moreover, we used two different definitions of metabolic health status to determine MUO/MHO phenotypes in a sample of both sexes (girls and boys) from all socioeconomic regions of a large central city in Iran. Furthermore, several potential confounders were adjusted in our analyses. Nevertheless, we acknowledge several limitations in our study. First, due to the cross-sectional design of the study, we were not able to infer a causal relation between AHEI-2010 and MUO/MHO. Therefore, conducting prospective studies is necessary. Second, due to the possible measurement errors and self-report nature of FFQ, misclassification of exposure could lead to unpredictable effects. Third, despite adjusting several covariates, residual confounders such as the state of puberty and sleep disorders might affect the relations. Finally, only adolescents who had overweight/obesity enrolled in our study; therefore, extrapolation our results to other adolescents should be done with caution.

## Conclusions

In conclusion, we found a strong adverse association between AHEI-2010 and odds of MUO profile in adolescents with overweight/obesity. Therefore, adherence to AHEI-2010 and its components could be an advisable approach for public health strategies in maintaining metabolic health among adolescents with overweight/obesity. Further studies, particularly with prospective nature, among different societies are required to confirm our results.

## Data Availability

The data that support the findings of this study are available from the corresponding author upon reasonable request.

## Abbreviations

AHEI: Alternative Healthy Eating Index
BP: Blood Pressure
BMI: Body Mass Index
CDC: Center for Disease Control
CI: Confidence Interval
DASH: Dietary Approaches to Stop Hypertension
DBP: Diastolic Blood Pressure
FBG: Fasting Blood Glucose
FFQ: Food Frequency Questionnaire
Hb A1C: Hemoglobin A1C
HDL-c: High-Density Lipoprotein cholesterol
HEI: Healthy Eating Index
HOMA-IR: Homeostasis Model Assessment Insulin Resistance
IDF: International Diabetes Federation
IR: Insulin Resistance
MetS: Metabolic Syndrome
MHO: Metabolically Healthy Overweight/Obesity
MUO: Metabolically Unhealthy Overweight/Obesity
OR: Odds Ratio
PA: Physical Activity
PAQ-A: Physical Activity Questionnaire for Adolescents
PUFA: Polyunsaturated Fatty Acids
SBP: Systolic Blood Pressure
SD: Standard Deviation
SE: Standard Error
SES: Socioeconomic Status
TG: Triglycerides
WC: Waist Circumference
WHO: World Health Organization
